# Supplementary material for: Cell-specific bioorthogonal tagging of glycoproteins
Source: Nat Commun. 2022 Oct 25;13:6237. doi: 10.1038/s41467-022-33854-0 (PMC9596482; doi:10.1038/s41467-022-33854-0)
Supplement: Supplementary file 1 — Supplementary Information [file 41467_2022_33854_MOESM1_ESM.pdf]

Supplementary Information: Cell-specific Bioorthogonal Tagging of Glycoproteins

B. Schumann *et al.*

Supplementary Figures

Supplementary Methods

Anna Cioce[1,2], Beatriz Calle[1,2,3], Tatiana Rizou[3,$], Sarah C. Lowery[4,$], Victoria L. Bridgeman[3,$], Keira E. Mahoney[4,$], Andrea Marchesi[1,2], Ganka Bineva-Todd[2], Helen Flynn[5], Zhen Li[1,2], Omur Y. Tastan[2], Chloe Roustan[6], Pablo Soro-Barrio[7], Mahmoud-Reza Rafiee[8], Acely Garza-Garcia[9], Aristotelis Antonopoulos[10], Thomas M. Wood[11,14], Tessa Keenan[12], Peter Both[13,15], Kun Huang[13,16], Fabio Parmeggian[13,17], Ambrosius P. Snijders[5], Mark Skehel[5], Svend Kjær[6], Martin A. Fascione[12], Carolyn R. Bertozzi[11], Stuart M. Haslam[10], Sabine L. Flitsch[13], Stacy A. Malaker[4], Ilaria Malanchi[3], Benjamin Schumann[1,2,*]

[1]Department of Chemistry, Imperial College London, W12 0BZ London, UK.

[2]Chemical Glycobiology Laboratory, The Francis Crick Institute, NW1 1AT London, UK.

[3]Tumour-Host Interaction Laboratory, The Francis Crick Institute, NW1 1AT London, UK.

[4]Department of Chemistry, Yale University, CT 06511 New Haven, USA.

[5]Proteomics Science Technology Platform, The Francis Crick Institute, NW1 1AT London, UK.

[6]Structural Biology Science Technology Platform, The Francis Crick Institute, NW1 1AT London, UK.

[7]Bioinformatics & Biostatistics Science Technology Platform, The Francis Crick Institute, NW1 1AT London, UK.

[8]RNA Networks Laboratory, The Francis Crick Institute, NW1 1AT London, UK.

[9]Mycobacterial Metabolism and Antibiotic Research Laboratory, The Francis Crick Institute, NW1 1AT London, UK.

[10]Department of Life Sciences, Imperial College London, SW7 2AZ London, UK.

[11]Sarafan ChEM-H, Department of Chemistry and Howard Hughes Medical Institute, Stanford University, CA 94305 Stanford, USA.

[12]Department of Chemistry, University of York, YO10 5DD York, UK.

[13]School of Chemistry & Institute of Biotechnology, The University of Manchester, M1 7DN Manchester, UK.
[14]current address: Massachusetts Institute of Technology, MA 02139 Cambridge, USA.
[15]current address: R&D Department, Axxence Slovakia s.r.o., 81107 Bratislava, Slovakia
[16]current address: Department of Chemistry and Biochemistry, University of Maryland, MD 20742 College Park, USA.
[17]current address: Department of Chemistry, Materials and Chemical Engineering "G. Natta", Politecnico di Milano, 20131 Milano, Italy
[$]These authors contributed equally.
*Correspondence should be addressed to: b.schumann@imperial.ac.uk.

Supplementary Figures

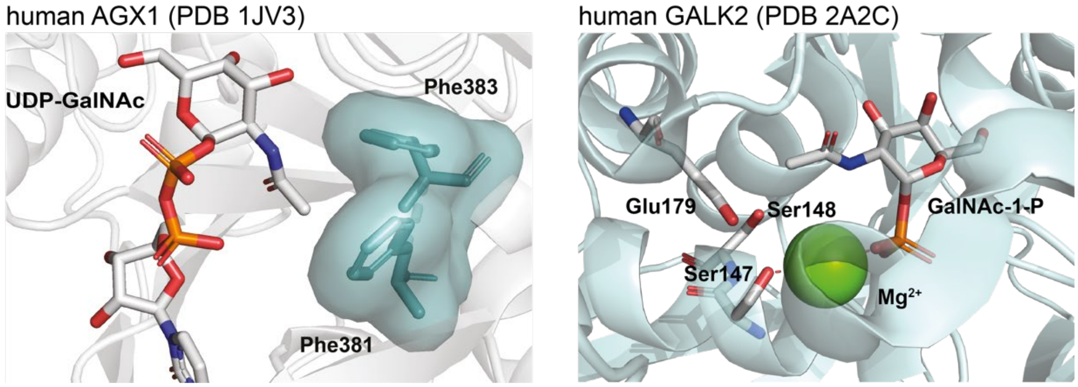

Supplementary Figure 1: Active site architectures of human enzymes of the GalNAc salvage pathway. In AGX1, the *N*-acyl side chain in UDP-GalNAc is in proximity to Phe381 and Phe383. In GALK2, the *N*-acyl side chain of GalNAc-1-phosphate is in proximity to the peptide backbone and amino acids forming a hydrogen network (Glu179, Ser147 and Ser148). Structures are modelled based on protein databank acquisition numbers 1JV3[1] and 2A2C.[2]

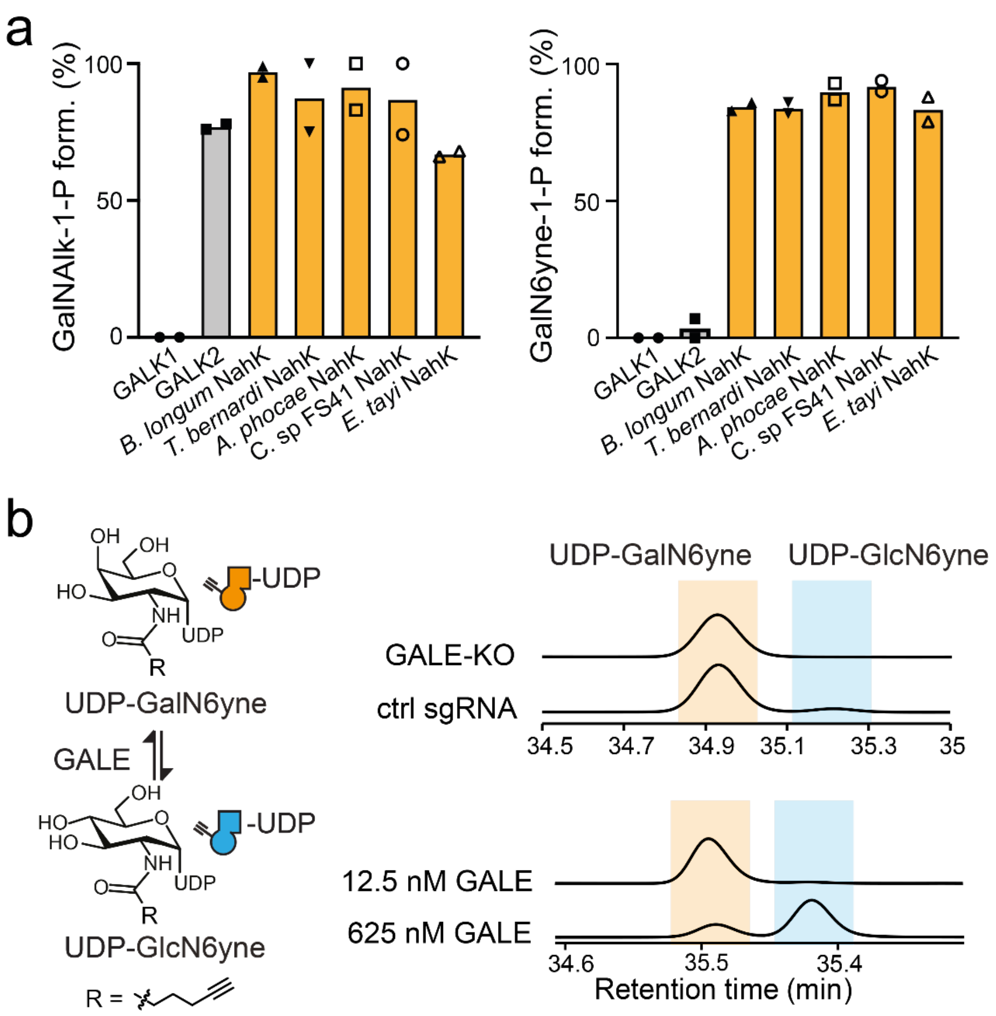

Supplementary Figure 2: Evaluation of enzymatic turnover of GalN6yne-based metabolites. a, *in vitro* GalN6yne-1-phosphate and GalNAlk-1-phosphate formation by human and bacterial GalNAc kinases, as assessed by Liquid Chromatography-Mass Spectrometry (LC-MS) and integrated ion count. Data are individual data points and means from two individual experiments. **b,** ion pair HPLC traces of *in vitro* epimerisation assays, using UDP-GalN6yne as substrate and either cell lysates from control or GALE-KO K-562 cells or two different concentrations of purified GALE as enzyme sources.[3] Data are representative of two independent replicates (lysate samples) or from one experiment (isolated GALE samples). Traces depict relative intensity of absorbance at 260 nm. Source data are provided as a Source Data file.

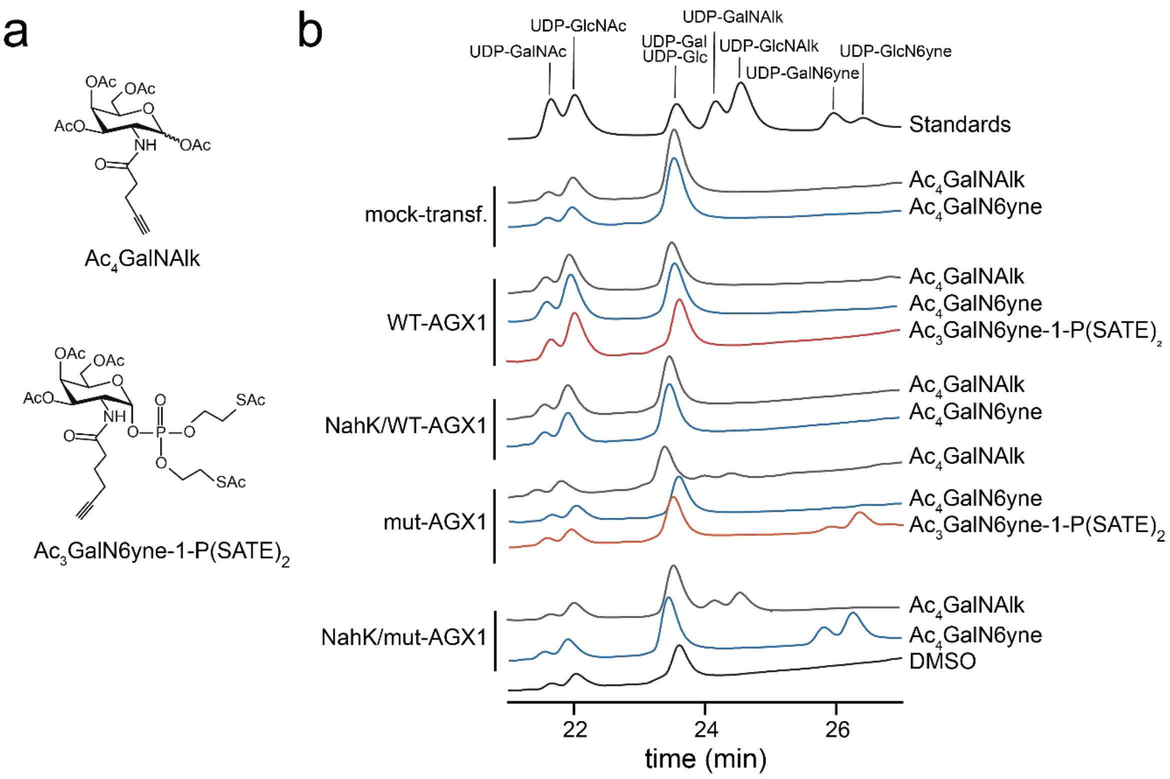

Supplementary Figure 3: Biosynthesis of chemically tagged UDP-sugars by metabolic engineering. a, structures of two MOE reagents used herein. Ac3GalN6yne-1-P(SATE)2 is a caged precursor of GalN6yne-1-phosphate. **b,** biosynthesis of UDP-sugars in cells mock or stably transfected with metabolic enzymes, as assessed by High Performance Anion Exchange Chromatography (HPAEC). Retention times were normalised on an external standards. Synthetic UDP-sugars served as standards. Data are from one representative out of two independent experiments performed on two different days. Source data are provided as a Source Data file.

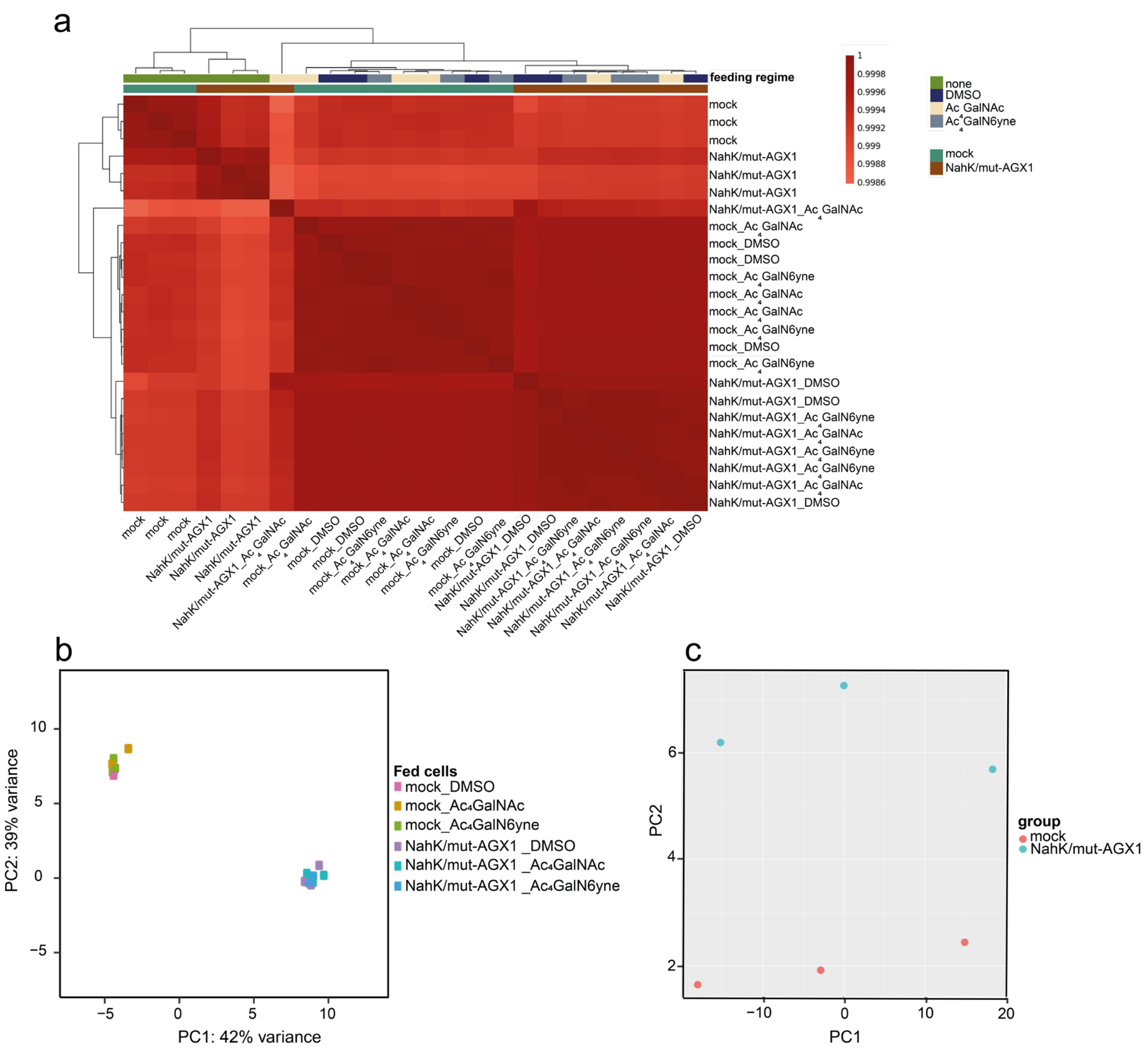

Supplementary Figure 4: Transcriptomic analysis of K-562 cells at different transfection conditions and feeding regimes. RNAs were extracted from K-562 cells transfected with either pSBbi-GH (mock) or pSBbi-NahK/mut-AGX1 at different feeding regimes (either unfed, DMSO, 10 μM Ac4GalNAc or 10 μM Ac4GalN6yne). **a,** Correlation plot and **b,** Principal component analysis (PCA) plot of transfected K-562 cells at different feeding regimes. Data are from one representative out of a total of three replicates collected on the same day. **c,** Previous experiment performed on RNA extracted from K-562 cells, transfected with either pSBbi-GH (mock) or pSBbi-NahK/mut-AGX1, showed significant variation in PCA associated with the different day of collection.

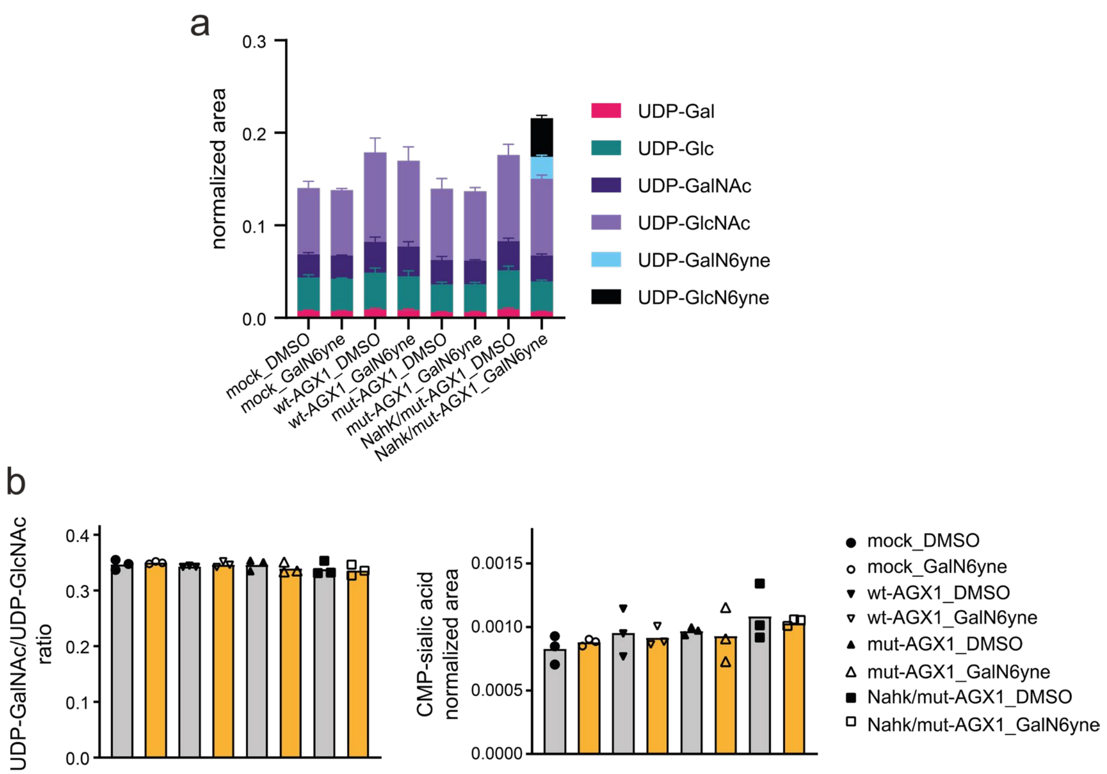

Supplementary Figure 5: Quantification of UDP-sugars assessed by High Performance Ion-pair Reversed-Phase Chromatography (IP RP HPLC). a, total amount of UDP-Hex, UDP-HexNAc and modified UDP-HexNAc in pSBbi-transfected K-562 cells fed with either 10 µM $Ac_4GalN6yne$ or DMSO. Data are from three biological replicates, collected on the same day, depicted as means + standard deviation (SD). **b,** UDP-GalNAc/UDP-GlcNAc ratio and CMP-sialic acid levels across the different K-562 cell lines and feeding regimes. Data are from three biological replicates, collected on the same day, depicted as individual data points and means. UDP-sugar areas were normalised against the total area between 0 and 65 min. Source data are provided as a Source Data file.

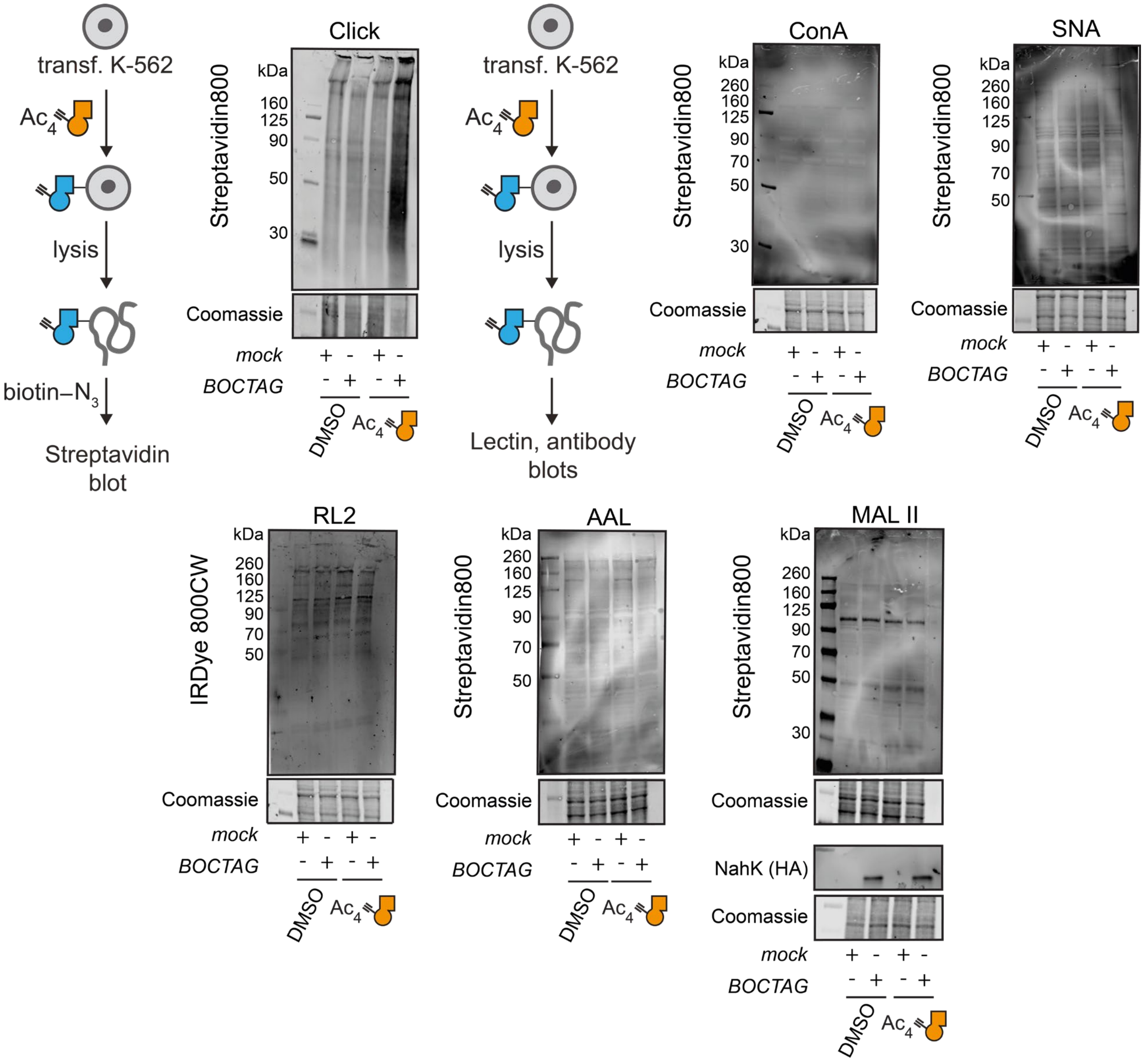

Supplementary Figure 6: Lectin and immunoblot analyses: BOCTAG and mock-transfected K-562 cells. Evaluation of whole lysate tagging and glycoproteins profile after treating K-562 stably expressing NahK/mut-AGX1 or an empty plasmid with either 10 µM Ac_4GalN6yne or DMSO. Data are from one experiment. ConA: Concanavalin A; AAL: Aleuria Aurantia Mushrooms; MAL II: Maackis Amurensis II, SNA: Sambucus Nigra; RL2: O-GlcNAc Antibody. Source data are provided as a Source Data file.

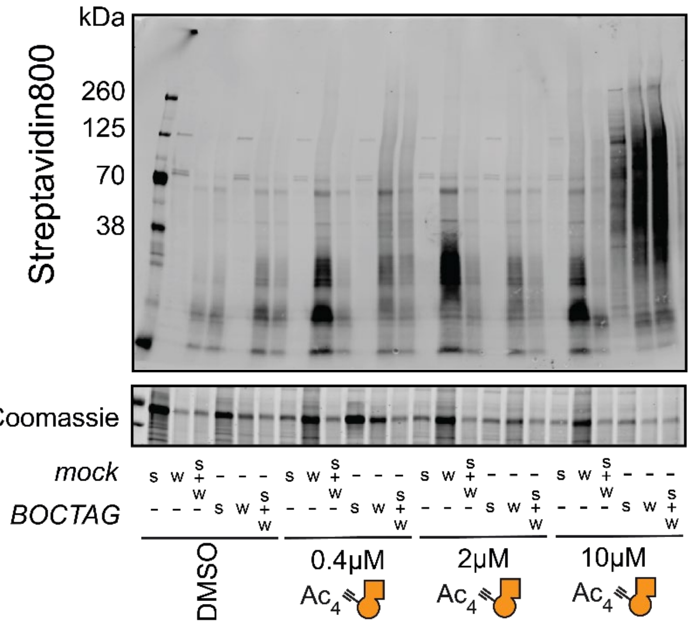

Supplementary Figure 7: Evaluation of chemical tag incorporation on cell surface, in cell lysate or both by feeding mock- and NahK/mut-AGX1-transfected K-562 cells with either different concentrations of Ac_4GalN6yne or DMSO. Glycoproteins were visualised by streptavidin blot after treating cells with biotin-picolyl azide under CuAAC conditions followed by streptavidin blot. Cell surface labelling was performed prior to cell lysis while whole lysate labelling was performed after lysis.

s: cell surface labelling, w: whole lysate labelling, s+w: cell surface and whole lysate labelling. Data are from one experiment. Source data are provided as a Source Data file.

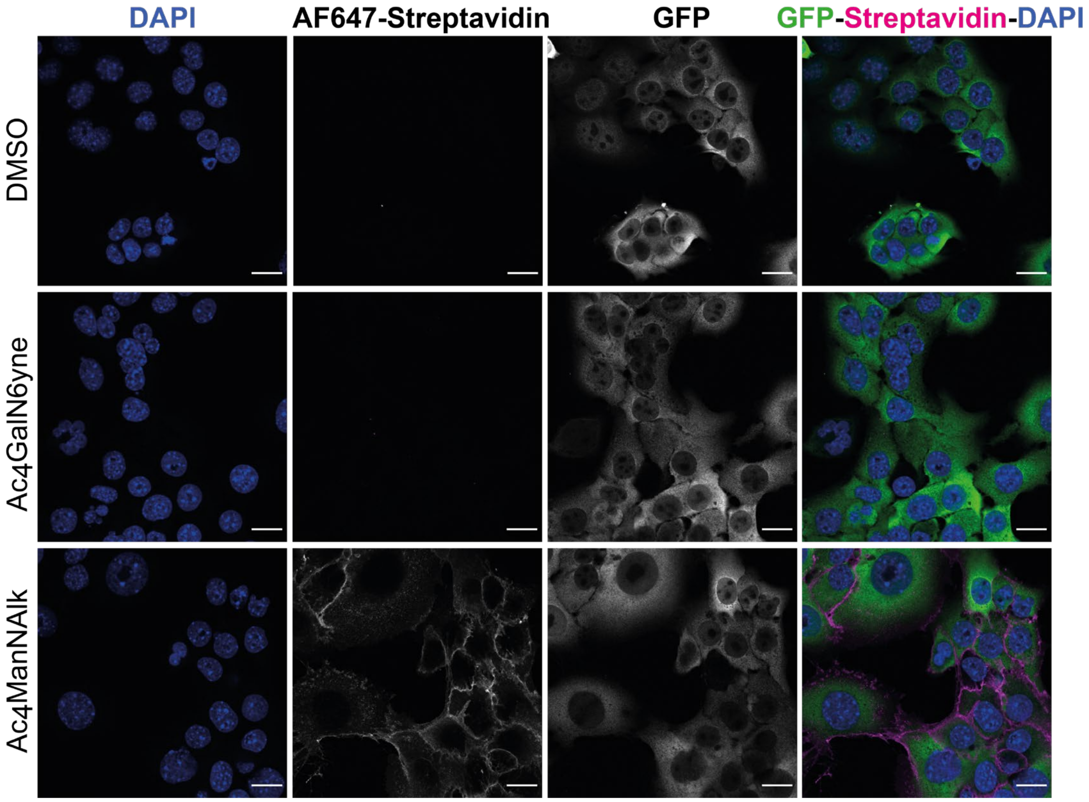

Supplementary Figure 8: Fluorescence microscopy of GFP-expressing 4T1 cells, transfected with empty plasmid pSBbi-Hyg, fed overnight with either DMSO, 50 µM $Ac_4GalN6yne$ or 50 µM $Ac_4ManNAlk$, treated with biotin-picolyl azide under CuAAC conditions and visualised with AlexaFluor647-Streptavidin. Scale bar, 20 µm. Data are representative of two independent experiments.

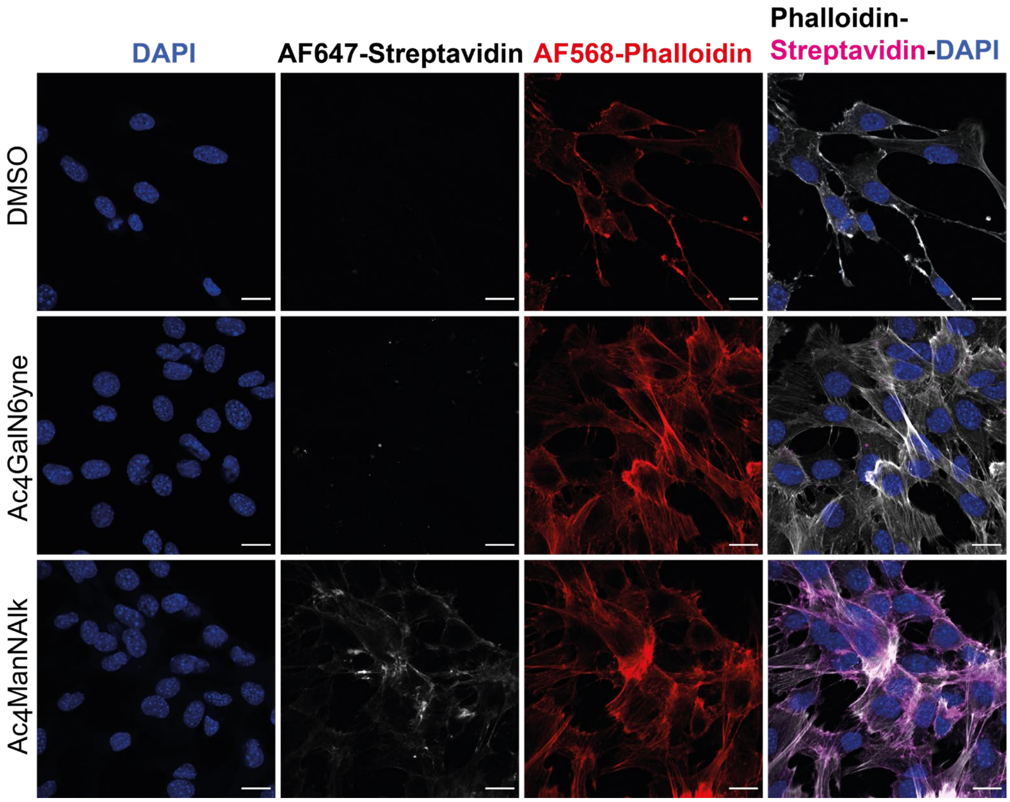

Supplementary Figure 9: Fluorescence microscopy of non-transfected MLg cells fed overnight with DMSO, 50 µM $Ac_4GalN6yne$ or 50 µM $Ac_4ManNAlk$ treated with biotin-picolyl azide under CuAAC conditions and visualised with AlexaFluor647-Streptavidin. Scale bar, 20 µm. Data are representative of two independent experiments.

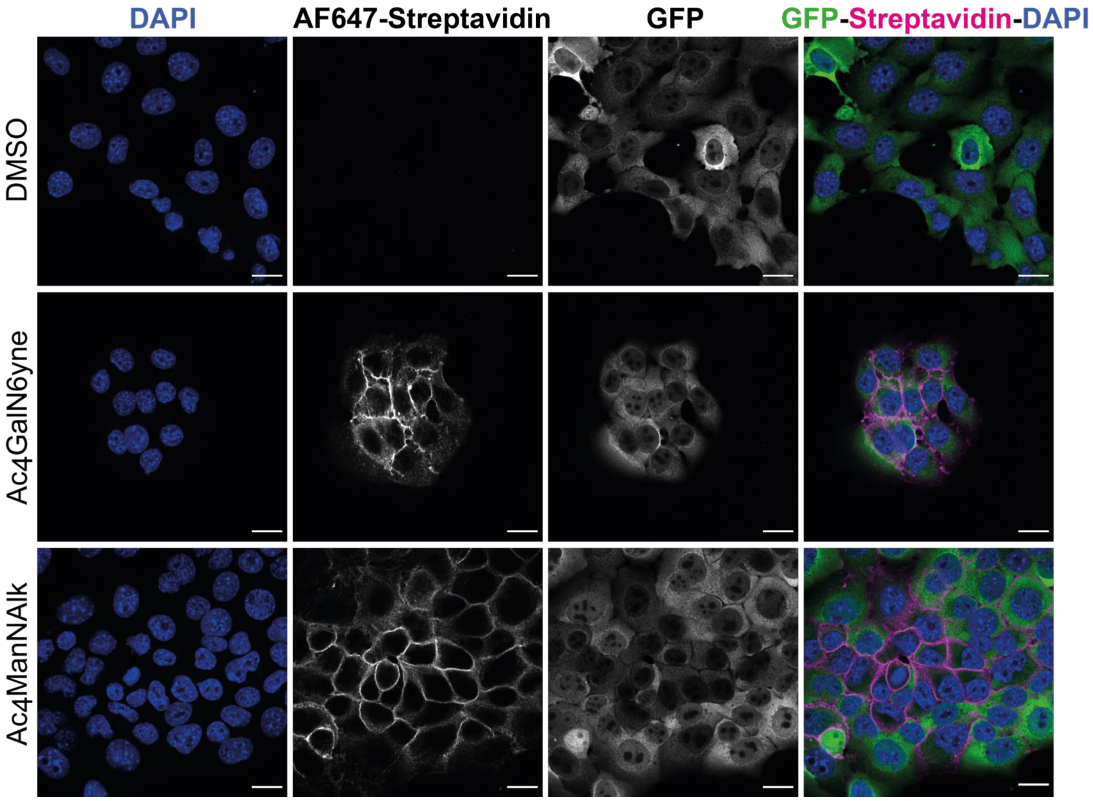

Supplementary Figure 10: Fluorescence microscopy of GFP-expressing 4T1 cells, transfected with pSBbi-NahK/mut-AGX1, fed overnight with either 50 µM Ac_4GalN6yne or 50 µM Ac_4ManNAlk treated with biotin-picolyl azide under CuAAC conditions and visualised with AlexaFluor647-Streptavidin. Scale bar, 20 µm. Data are representative of two independent experiments.

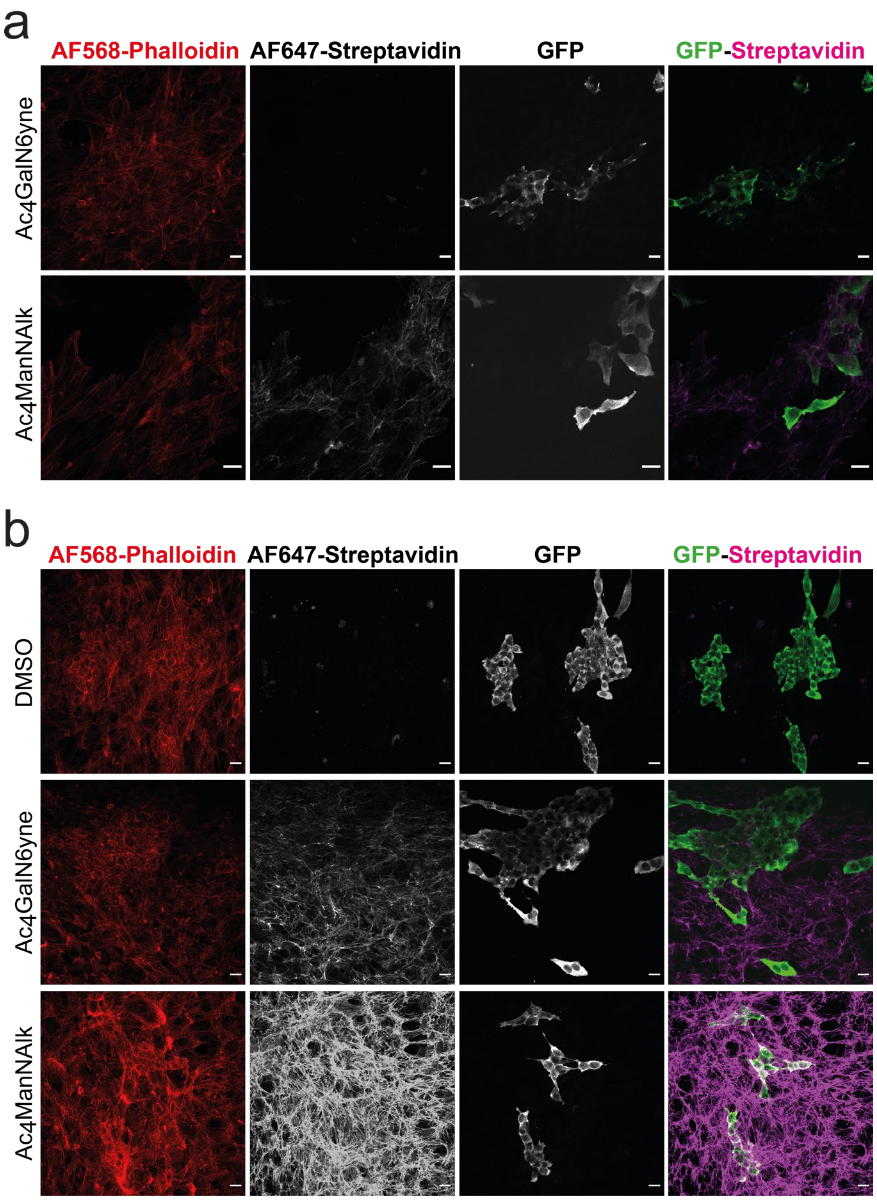

Supplementary Figure 11: Maximum intensity projection from a z-stack acquisition of **a,** GFP-expressing 4T1 and MLg cells, both transfected with pSBbi-Hyg empty plasmid, in a co-culture system fed overnight with either 50 µM $Ac_4GalN6yne$ or 50 µM $Ac_4ManNAlk$. **b,** GFP-expressing 4T1 and MLg cells, both transfected with pSBbi-NahK/mut-AGX1 plasmid, in a co-culture system fed overnight with either DMSO, 50 µM $Ac_4GalN6yne$ or 50 µM $Ac_4ManNAlk$. Co-culture samples in both a and b were treated with biotin-picolyl azide under CuAAC conditions and visualised by AlexaFluor647-Streptavidin. Scale bar, 20 µm. Data are representative of two independent experiments.

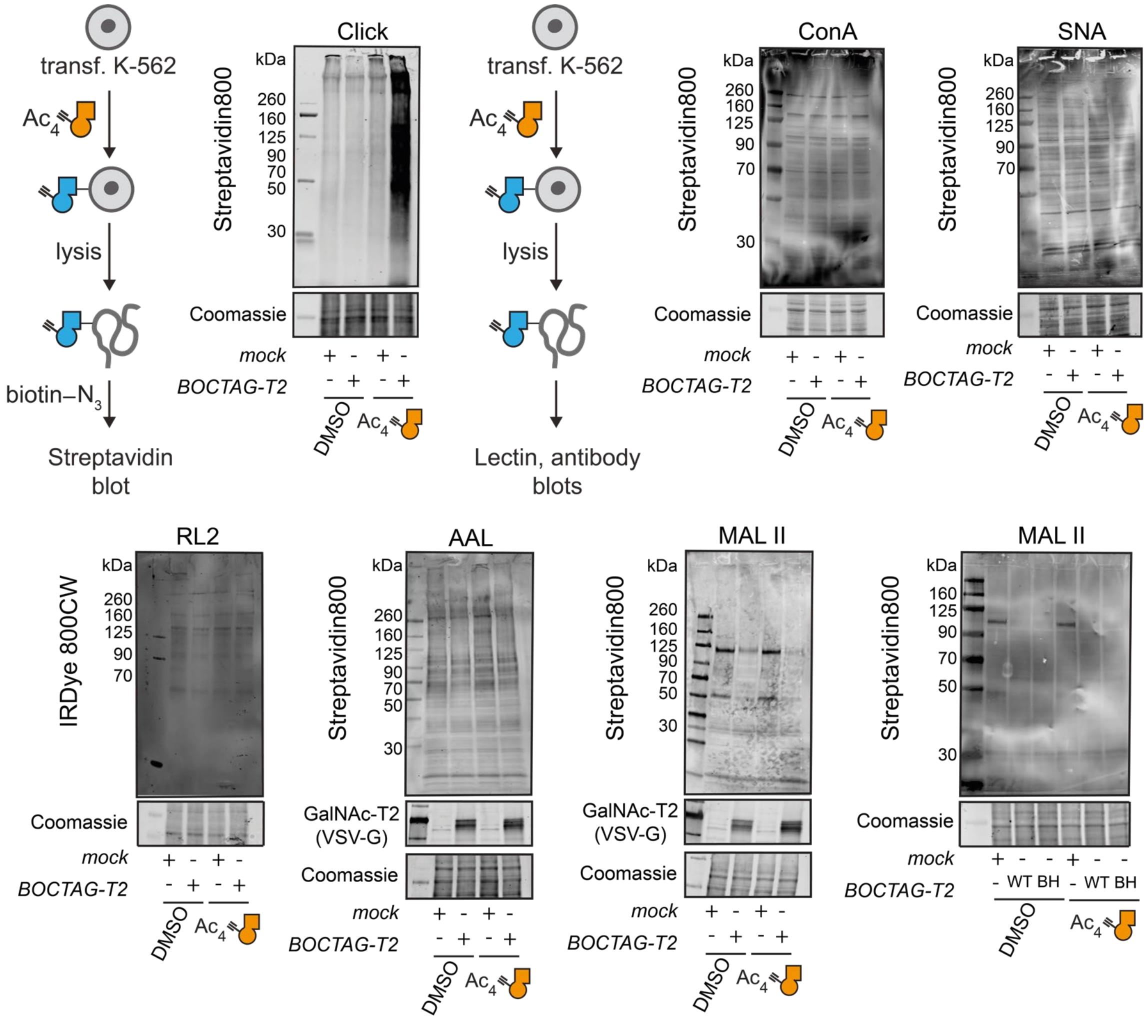

Supplementary Figure 12: Lectin and immunoblot analyses: BOCTAG-T2 and mock-transfected K-562 cells. Evaluation of whole lysate tagging and glycoproteins profile after treating K-562 stably expressing NahK/mut-AGX1/BH-T2 or an empty plasmid with either 10 µM Ac4GalN6yne or DMSO. ConA: Concanavalin A; AAL: Aleuria Aurantia Mushrooms; MAL II: Maackis Amurensis II, SNA: Sambucus Nigra; RL2: O-GlcNAc Antibody. Data are from one experiment. Source data are provided as a Source Data file.

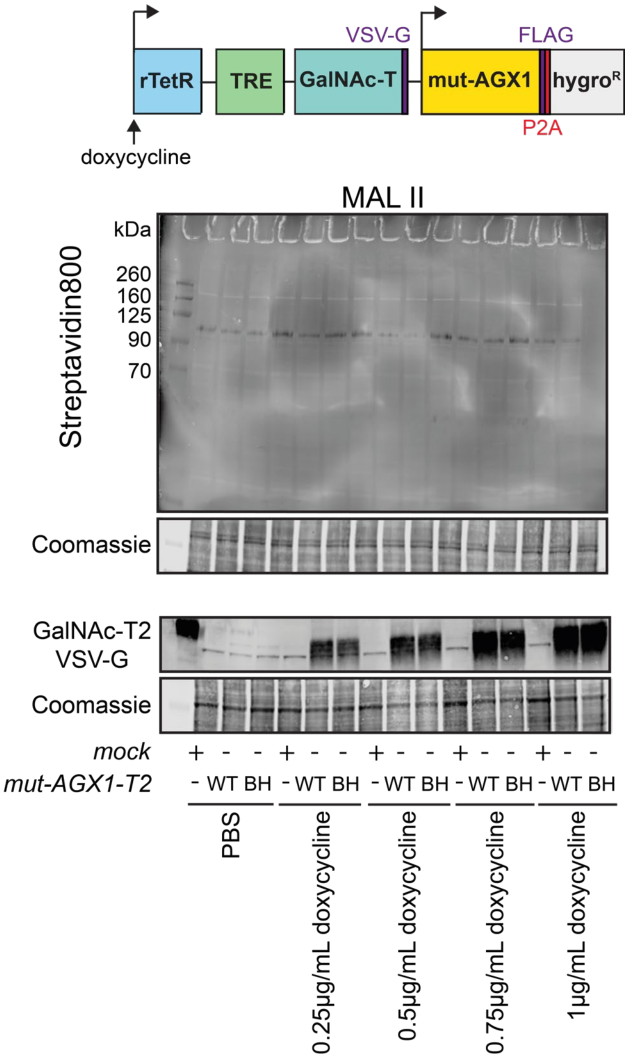

Supplementary Figure 13: MAL II binding at different WT- or BH-GalNAc-T2 expression levels. Cloning WT- or BH-GalNAc-T2 in a plasmid with a tetracycline-dependent promoter (pSBtet) allows to induce and modulate the expression levels of those enzymes in presence of tetracycline or one of its analogues like doxycycline. MAL II blot shows a change in the binding profile between mock- and mut-AGX1-T2 by increasing the expression levels of GalNAc-T2 (for both WT- and BH-GalNAc-T2 enzymes). Dose-response of WT- or BH-GalNAc-T2 expression is evaluated by anti-VSV-G staining. Data are from one experiment. Source data are provided as a Source Data file.

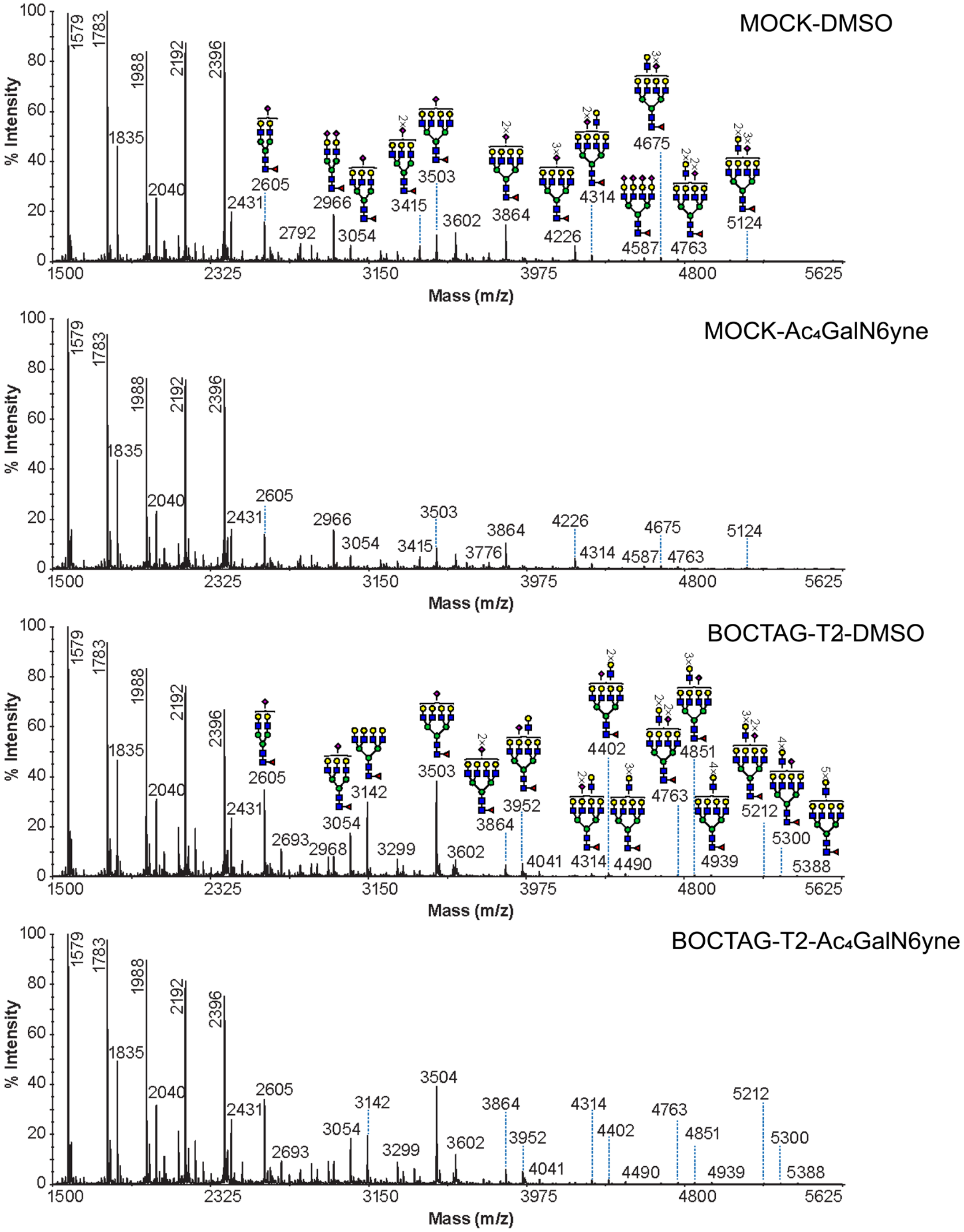

Supplementary Figure 14: Overall N-glycan structural analysis of K-562 cells transfected with mock or NahK/mut-AGX1-BH-GalNAc-T2 plasmids treated with either 10μM Ac4GalN6yne or DMSO. N-glycans were released by peptide N-glycosidase F digestion, permethylated and analysed by MALDI-TOF/TOF. Data are from one experiment.

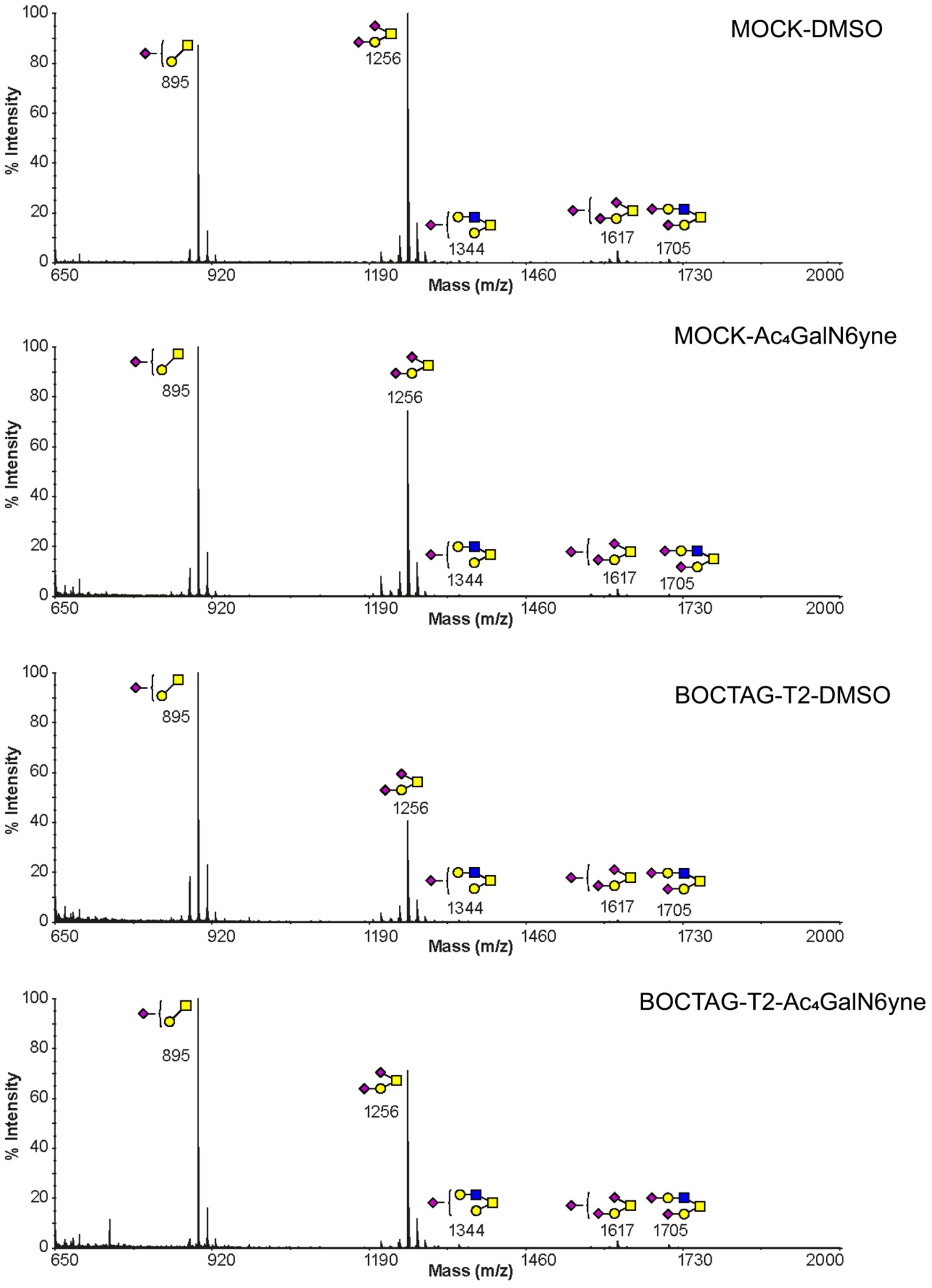

Supplementary Figure 15: Overall O-glycan structural analysis of K-562 cells transfected with mock- or NahK/mut-AGX1-BH-GalNAc-T2 plasmids treated with either 10µM $Ac_4GalN6yne$ or DMSO. Data are from one experiment. O-glycans were released by reductive elimination, permethylated and analysed by MALDI-TOF/TOF. Data are from one experiment.

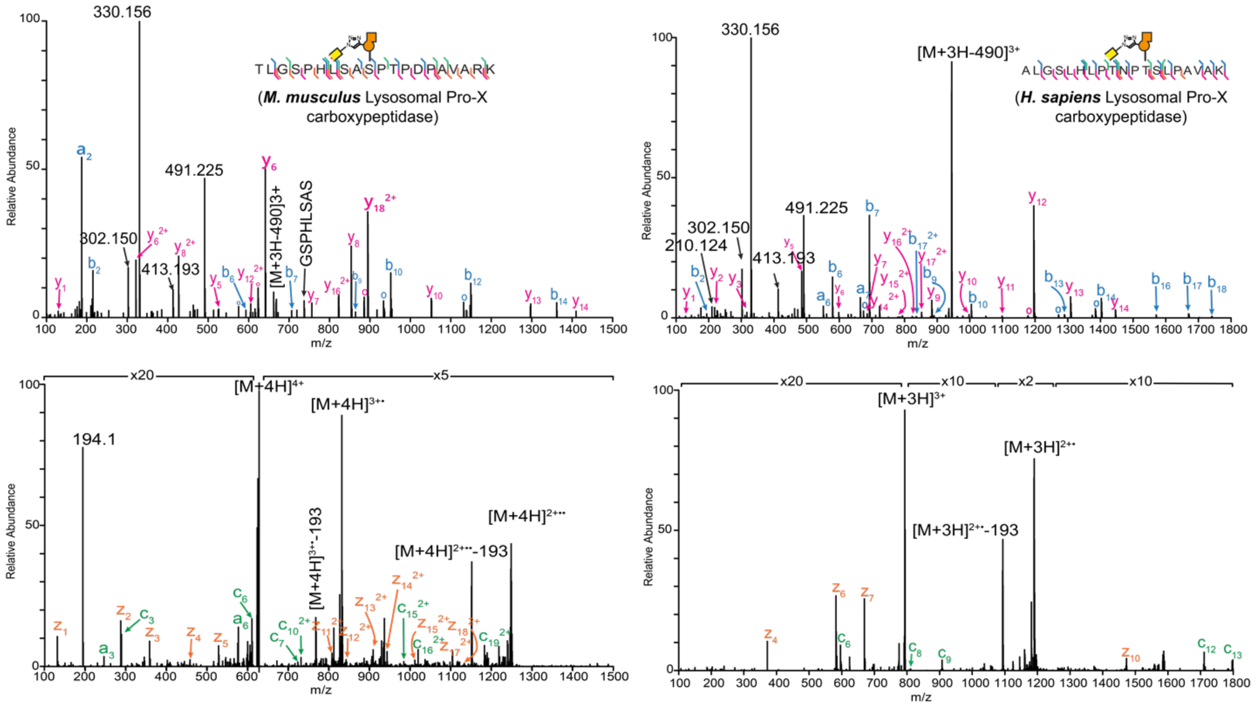

Supplementary Figure 16. Higher-energy Collisional Dissociation (HCD) and Electron Transfer Dissociation (ETD) spectra of homologous glycopeptides from murine (left) and human (right) origins enriched in secretome (**Fig. 5**).

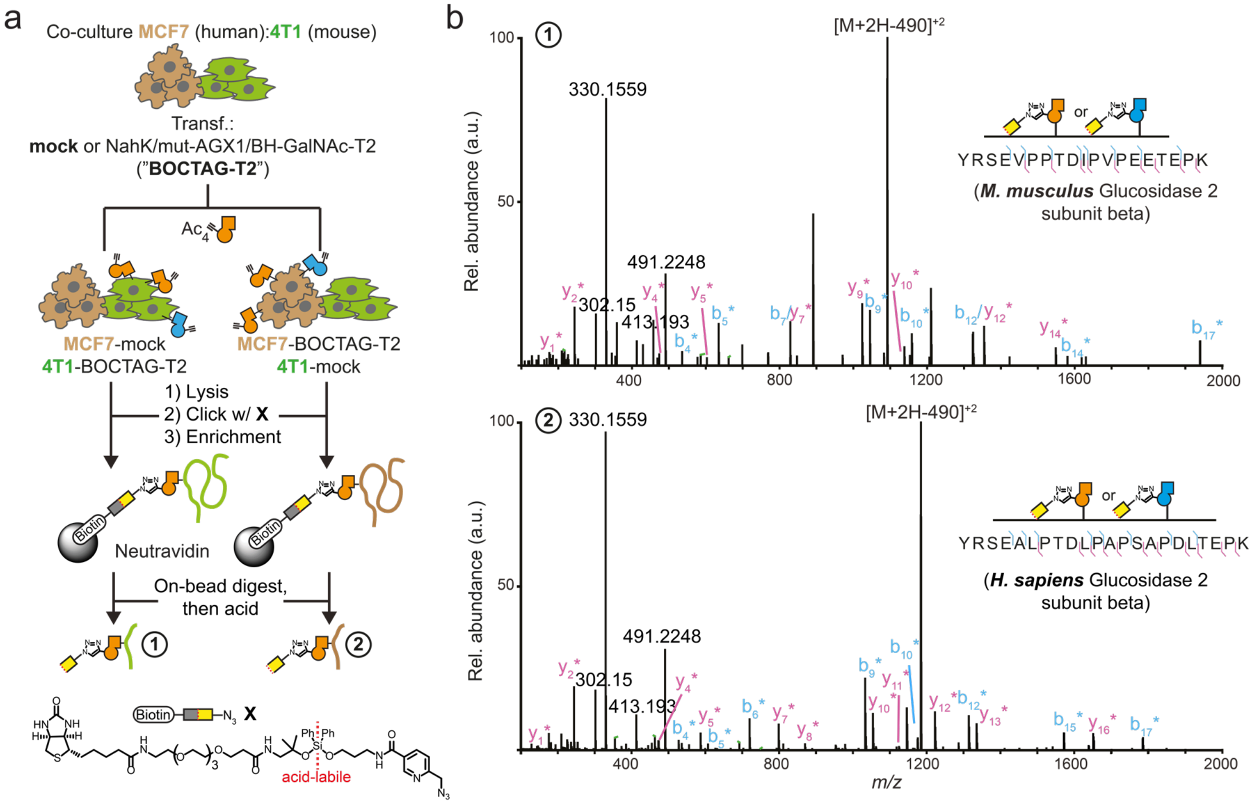

Supplementary Figure 17: Glycoproteomic analysis of lysates of co-cultured human and murine samples. a, cell-selective enrichment and MS-glycoproteomics of murine-human co-culture systems. MCF7 and 4T1 cells transfected as indicated were co-cultured overnight and treated with DMSO or 10 µM Ac4GalN6yne for 24h. Lysates were subjected to CuAAC with acid-cleavable biotin-picolyl azide **X** and enriched on neutravidin beads. On-bead digest yielded peptide fractions while acid treatment of beads yielded glycopeptide fractions. **b,** Higher-energy Collisional Dissociation (HCD) spectra of homologous glycopeptides from murine (top) and human (bottom) origins.

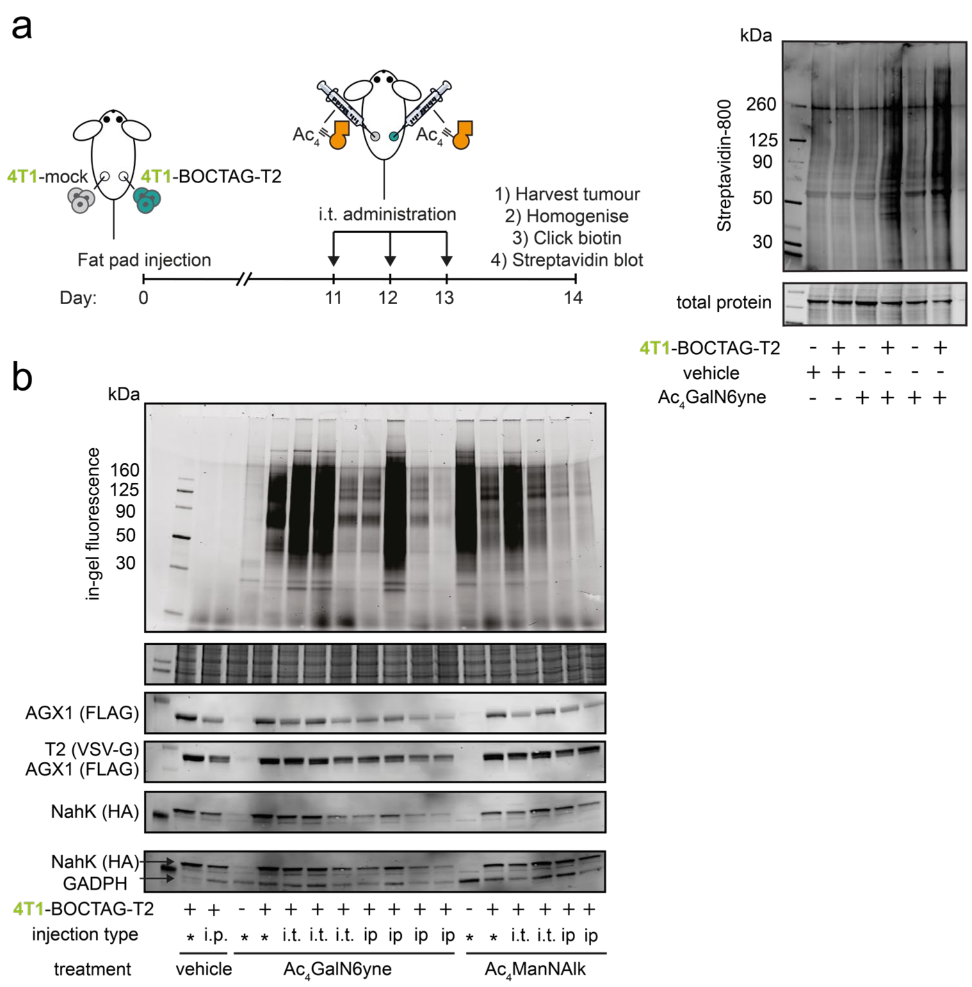

Supplementary Figure 18: BOCTAG labels glycoproteins in a cell-specific manner *in vivo*. **a,** BOCTAG-T2 and mock tumours were grown in the same mouse treated systemically for three days with 15 mg/kg Ac4GalN6yne (n=2) or the corresponding volume of vehicle (5% (v/v) DMSO/ PEG-400, n=1) by intratumoral (i.t.) injection. Tumours were harvested, lysed, subjected to CuAAC with biotin-picolyl azide and analysed by streptavidin blot. **b,** after the experiment in **Fig.5** and **Supplementary Figure 18a**, BOCTAG-T2 cells were collected from intratumoral (i.t.) and intraperitoneal (i.p.) treated tumours, plated for 10 days in DMEM growing media and fed with either DMSO, Ac4GalN6yne or Ac4ManNAlk. Cell surface glycoprotein labelling efficiency of primary cells was validated by CuAAC with CF680-picolyl azide. Western blot was performed to assess levels of expression of BOCTAG-T2 enzymes. i.p.= intraperitoneal injection, i.t.= intratumoral injection, * = parental 4T1-mock and 4T1-BOCTAG-T2 cell lines. Data is representative of two independent experiments. Source data are provided as a Source Data file.

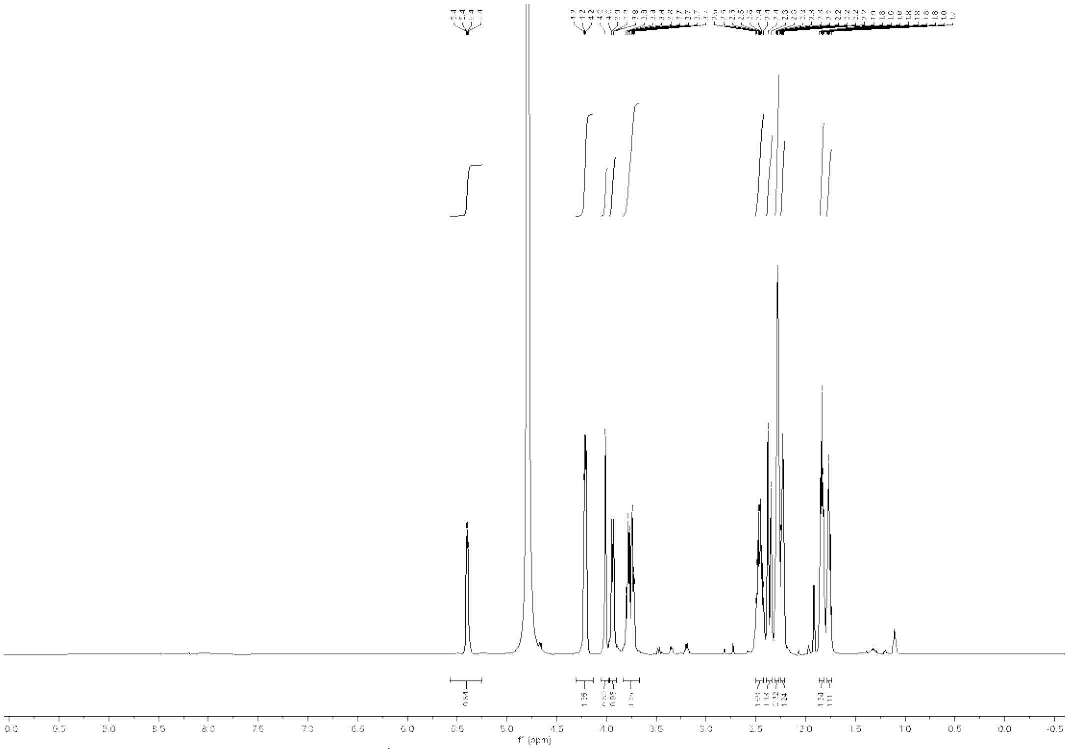

Supplementary Figure 19. ^{1}H NMR (600 MHz, D_2O) of 2-deoxy-2-(5-hexynoyl)amido-α-D-galactopyranosyl phosphate disodium salt **SI-3**

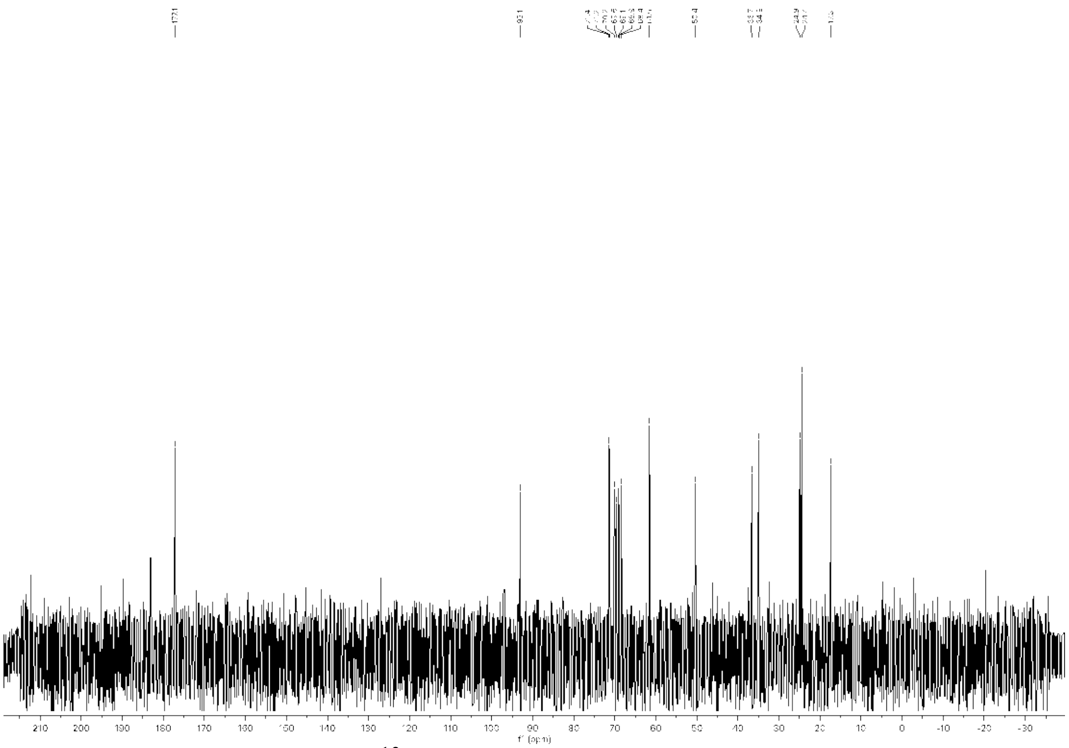

Supplementary Figure 20. ^{13}C NMR (150 MHz, D_2O) of 2-deoxy-2-(5-hexynoyl)amido-α-D-galactopyranosyl phosphate disodium salt **SI-3**

Supplementary Methods

Chemical synthesis

2-deoxy-2-(5-hexynoyl)amido-α-D-galactopyranosyl phosphate disodium salt (SI-3)

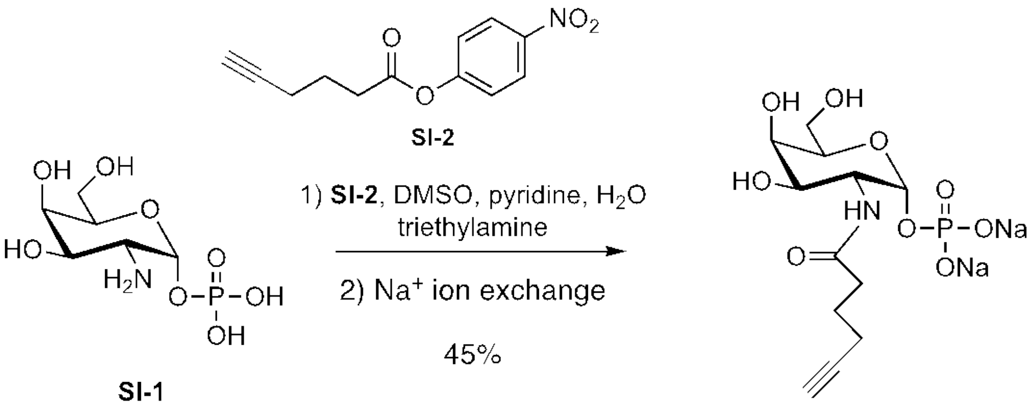

GalN6yne-1-phosphate (**SI-3**)

Commercial amine **SI-1** (15 mg, 58 µmol) in anhydrous DMSO/pyridine (1:1.5 (v/v), 1.2 mL) was treated with 4-nitrophenyl hex-5-ynoate **SI-2** (20 mg, 87 µmol) and triethylamine (8 µL, 58 µmol). The turbid solution was left to stir overnight. The reaction was treated with 100 µL water and left to stir for another 8 h. Another 150 µL water were added as well as 1.5 equiv. reagent **SI-2** (20 mg, 87 µmol). After another 60 h, another 200 µL water were added, then another 1.5 equiv. reagent **SI-2** (20 mg, 87 µmol). The yellow solution turned clear overnight, when TLC (DCM/MeOH/water 4:1:1 with 2 drops triethylamine) indicated conversion. The solution was shock frozen on dry ice, lyophilised and purified by size exclusion chromatography (Sephadex G-25 extra fine, Sigma-Aldrich) using water/MeOH 3:2 (v/v) as a solvent. The combined fractions were concentrated and passed through AG 50W-X8 Na^+ form resin (Bio-Rad) and lyophilised to give alkyne **SI-3** (10 mg, 26 µmol, 45%) as a white solid. ^{1}H NMR (600 MHz, D_2O) δ 5.40 (dd, J = 7.5, 3.5 Hz, 1H), 4.31 – 4.14 (m, 2H), 4.01 (s, 1H), 3.94 (dd, J = 10.9, 3.1 Hz, 1H), 3.84 – 3.67 (m, 2H), 2.46 (m, J = 14.8, 7.2 Hz, 2H), 2.39 – 2.33 (m, 2H), 2.28 (m, 4H), 2.23 (m, 1H), 1.84 (m, 1H), 1.76 (m, 1H); ^{13}C NMR (150 MHz, D_2O) δ 177.1, 93.1, 71.4, 71.2, 70.2, 69.6, 69.1, 68.9, 68.4, 61.6, 50.4, 36.7, 34.9, 24.9, 24.4, 17.3; HRMS (m/z): $[M-H]^-$ calcd. for $C_{12}H_{20}NO_9P$, 352.0876; found, 352.0800.

Supplementary References

1. Pennef, C. *et al.* Crystal strucutre of two human pyrophosphorylase isoforms in complexes with UDPGlc(Gal)NAc: Role of the alternatively spliced insert in the enzyme oligomeric assembly and active site architecture. *EMBO J.* **20**, 6191–6202 (2001).

2. Thoden, J. B. & Holden, H. M. The molecular architecture of human N-acetylgalactosamine kinase. *J. Biol. Chem.* **280**, 32784-32791 (2005).

3. Debets, M. F. *et al.* Metabolic precision labeling enables selective probing of O-linked N-acetylgalactosamine glycosylation. *Proc. Natl. Acad. Sci.* **117**, 25293-25301 (2020).
