# Supplementary material for: Cell-specific bioorthogonal tagging of glycoproteins
Source: Nat Commun. 2022 Oct 25;13:6237. doi: 10.1038/s41467-022-33854-0 (PMC9596482; doi:10.1038/s41467-022-33854-0)
Supplement: Supplementary file 3 — Description of Additional Supplementary Files [file 41467_2022_33854_MOESM3_ESM.docx]

Description of additional supplementary files

- File name: Supplementary data 1

Description: list of glycoproteins selectively enriched by cells expressing NahK/mut-AGX1 or NahK/mut-AGX1-BH-T2 in SILAC-based proteomics experiment

- File name: Supplementary data 2

Description: list of peptides and glycopeptides selectively enriched by cells expressing NahK/mut-AGX1-BH-T2 in murine VS human co-culture samples (from secretome).

- File name: Supplementary data 3

Description: list of glycopeptides selectively enriched by cells expressing NahK/mut-AGX1-BH-T2 in murine VS human co-culture samples (from lysate).

- File name: Supplementary data 4

Description: sequences of oligonucleotides
