# Supplementary material for: Cell-specific bioorthogonal tagging of glycoproteins
Source: Nat Commun. 2022 Oct 25;13:6237. doi: 10.1038/s41467-022-33854-0 (PMC9596482; doi:10.1038/s41467-022-33854-0)
Supplement: Supplementary file 8 — Reporting Summary [file 41467_2022_33854_MOESM8_ESM.pdf]

nature portfolio

Corresponding author(s): Benjamin Schumann

Last updated by author(s): Oct 3, 2022

Reporting Summary

Nature Portfolio wishes to improve the reproducibility of the work that we publish. This form provides structure for consistency and transparency in reporting. For further information on Nature Portfolio policies, see our Editorial Policies and the Editorial Policy Checklist.

Statistics

For all statistical analyses, confirm that the following items are present in the figure legend, table legend, main text, or Methods section.

n/a	Confirmed	
☐	☒	The exact sample size (*n*) for each experimental group/condition, given as a discrete number and unit of measurement
☐	☒	A statement on whether measurements were taken from distinct samples or whether the same sample was measured repeatedly
☐	☒	The statistical test(s) used AND whether they are one- or two-sided<br>*Only common tests should be described solely by name; describe more complex techniques in the Methods section.*
☒	☐	A description of all covariates tested
☐	☒	A description of any assumptions or corrections, such as tests of normality and adjustment for multiple comparisons
☐	☒	A full description of the statistical parameters including central tendency (e.g. means) or other basic estimates (e.g. regression coefficient) AND variation (e.g. standard deviation) or associated estimates of uncertainty (e.g. confidence intervals)
☐	☒	For null hypothesis testing, the test statistic (e.g. *F*, *t*, *r*) with confidence intervals, effect sizes, degrees of freedom and *P* value noted<br>*Give P values as exact values whenever suitable.*
☒	☐	For Bayesian analysis, information on the choice of priors and Markov chain Monte Carlo settings
☒	☐	For hierarchical and complex designs, identification of the appropriate level for tests and full reporting of outcomes
☒	☐	Estimates of effect sizes (e.g. Cohen's *d*, Pearson's *r*), indicating how they were calculated

Our web collection on statistics for biologists contains articles on many of the points above.

Software and code

Policy information about availability of computer code

Data collection

UPLC (Ultra Performance Liquid Chromatography)-MS was controlled by MassLynx Spectrometry Software.

High Performance Anion Exchange Chromatography (HPAEC) was controlled by Chromeleon Chromatography Data System (CDS).

High Performance Ion-pair reversed-phase Chromatography (IP RP HPLC) was controlled by Agilent OpenLab CDS, ChemStation Edition Rev. C.01.10[239]

Odyssey CLx imager was controlled by ImageStudio Pro software.

Orbitrap Fusion Lumos with ETD (Electron Transfer Dissociation Mass Spectrometry, Thermo Fisher) and Orbitrap Eclipse with ETD, both coupled to an UltiMate 3000 RSLCnano, were controlled by Xcalibur software.

Zeiss LSM710 Invert microscope was controlled by Zen software.

Data analysis

Data acquired on UPLC were analysed by MassLynx Spectrometry Software.
MS and MS/MS were processed by using Data Explorer 4.9 Software (Applied Biosystems).
SILAC-based proteomics raw data were processed by Maxquant (version 1.6.5.0) and R programming environment (version 4.1.3).
LFQ-based proteomics raw data were processed by Maxquant (version 1.6.5.0) and Perseus software (version 4.1.3).
LFQ-based glycoproteomics data are analysed by Byonic™ software (version 4.0.12).
In-gel fluorescence and Western Blot analysis were analysed by ImageStudio lite (version 5.2).
light microscopy images were analysed by Zen and fiji software.
Adobe Photoshop 2022 was used to crop gels.
Adobe illustrator 2022 was used to assemble Figures and re-label plot axes.
GraphPad Prism 9.1.0(221) was used to make bar charts and plots

NMR data were analysed by Mestrenova (version 14.2.2)

In RNA transcriptomics analysis, cutadapt (version 1.5) was used for trimmering raw data, STAR (version 2.5.2) was used to perform alignment, RSEM (version 1.3.0) was used to obtain gene abundance, R programming environment (version 3.6.1) was used to analyse the data with packages and tools that has available. DESeq2 package (version 1.24.0)-R package was used to normalize raw count data and perform differential expression.

For manuscripts utilizing custom algorithms or software that are central to the research but not yet described in published literature, software must be made available to editors and reviewers. We strongly encourage code deposition in a community repository (e.g. GitHub). See the Nature Portfolio guidelines for submitting code & software for further information.

Data

Policy information about availability of data

All manuscripts must include a data availability statement. This statement should provide the following information, where applicable:

- Accession codes, unique identifiers, or web links for publicly available datasets
- A description of any restrictions on data availability
- For clinical datasets or third party data, please ensure that the statement adheres to our policy

Mass spectrometry proteomics and glycoproteomics data generated in this study have been deposited in the PRIDE database under accession code PXD035430 [http://www.ebi.ac.uk/pride/archive/projects/PXD035430], PXD035437 [http://www.ebi.ac.uk/pride/archive/projects/PXD035437], PXD035438 [http://www.ebi.ac.uk/pride/archive/projects/PXD035438], PXD035445 [http://www.ebi.ac.uk/pride/archive/projects/PXD035445] and PXD035449 [http://www.ebi.ac.uk/pride/archive/projects/PXD035449]
Mass spectrometry Glycomics data generated in this study have been deposited in the GlycoPost database under accession code GPST000293 [https://glycopost.glycosmos.org/entry/GPST000293.0].
RNA-sequencing data generated in this study have been deposited in the GEO database under accession code GSE213052 [https://www.ncbi.nlm.nih.gov/geo/query/acc.cgi?acc=GSE213052].
The experiment data that support the finding of this study are available from the corresponding author upon request without any reservation.

For proteomics and glycoproteomics data, Homo sapiens and Mus musculus FASTA protein sequences databases from UniProt were used as reference database. While for RNA trancriptomics analysis, gene levels were counted against the human genome GRCh38 from NCBI.

Field-specific reporting

Please select the one below that is the best fit for your research. If you are not sure, read the appropriate sections before making your selection.

☒ Life sciences ☐ Behavioural & social sciences ☐ Ecological, evolutionary & environmental sciences

For a reference copy of the document with all sections, see nature.com/documents/nr-reporting-summary-flat.pdf

Life sciences study design

All studies must disclose on these points even when the disclosure is negative.

Sample size

Sample size was chosen based on previous experiments and standards. Effect sizes for experiments in vitro and in living cells were estimated based on previous reports (Schumann et al., Mol. Cell 2020). Effect sizes for in vivo experiments were estimated based on experience from in vitro work. Specifically, a 10-fold increase in streptavidin signal was expected (10+-4 vs 1+-2 a.u.), thus n = 3 with alpha = 0.05 and 90% probability.

In-gel fluorescence and western blot experiments were designed included both negative and positive controls, with at least two independent replicates for most experiments. Experiments with quantification were performed in at least three independent replicates.
In all the proteomics and glycoproteomics experiments, combinations of cell type and feeding conditions were chosen according to the preliminary in-gel and western blot data. SILAC experiments were combined in forward and reverse conditions.

In vitro enzymatic assays were performed in at least duplicate including positive controls.

Data exclusions

no data were excluded from the analysis.

Replication

in vitro enzymatic reactions were performed at least in duplicates.
Light microscopy experiment were performed in duplicate.
SILAC-based proteomics analysis were performed in triplicates.
in-gel fluorescence or western blot analysis were performed at least in duplicate.
HPAEC-based analysis were performed in duplicate.
All attempts of replication were successful and gave similar results.

Randomization

Randomization of animals was not required because mice were treated with one tumour each from control and BOCTAG cell lines.

Blinding

Experiments were performed non-blinded because both control and BOCTAG tumours were grown in the same mouse, hence no bias due to e.g. housing conditions was expected.

Reporting for specific materials, systems and methods

We require information from authors about some types of materials, experimental systems and methods used in many studies. Here, indicate whether each material, system or method listed is relevant to your study. If you are not sure if a list item applies to your research, read the appropriate section before selecting a response.

Materials & experimental systems

n/a	Involved in the study
☐	☒ Antibodies
☐	☒ Eukaryotic cell lines
☒	☐ Palaeontology and archaeology
☐	☒ Animals and other organisms
☒	☐ Human research participants
☒	☐ Clinical data
☒	☐ Dual use research of concern

Methods

n/a	Involved in the study
☒	☐ ChIP-seq
☒	☐ Flow cytometry
☒	☐ MRI-based neuroimaging

Antibodies

Antibodies used

rabbit anti-FLAG (PA1-984B, Invitrogen) used at1:1000 in WB
rabbit anti-HA (ab9110, Abcam) used at 1:1000 in WB
goat anti-VSV-G (ab3861, Abcam) used at 1:2000 in WB
goat anti-GFP (ab5450, Abcam) used at1 :300 dilution in light microscopy assays.
rabbit anti-GADPH (ab181602, Abcam) used at 1:5000 in WB
rabbit anti-VSV-G (PA129903, Invitrogen) used at 1:500 in WB
mouse anti-RL2 (ab2739, Abcam) used at 1:500 in WB

Validation

goat anti-GFP was validated on GFP-expressing 4T1 (murine breast cancer) using GFP-free MLg (murine fibroblast) cells in both mono- and co-culture samples in light microscopy experiment.

All the other antibodies were validated in western blot assays by including positive and negative controls of k562 transfected-cells.

Eukaryotic cell lines

Policy information about cell lines

Cell line source(s)

K-562 (ATCC CCL-243), 4T1 (ATCC CRL-2539), MCF7 (ATCC HTB-22), MLg (ATCC CCL-206), SF21 (ATCC CRL-1711)

Authentication

all the cells used in the manuscript were authenticated by The Francis Crick Institute Cell Services STP by STR profiling (for human lines) and species identification for validation.

Mycoplasma contamination

all cell lines tested negative for mycoplasma contamination at the Francis Crick Institute Cell Services STP

Commonly misidentified lines
(See ICLAC register)

none

Animals and other organisms

Policy information about studies involving animals; ARRIVE guidelines recommended for reporting animal research

Laboratory animals

NOD-SCID IL2Rgnull (NSG) strain mice (strain nomenclature NOD.Cg-PrkdcSCID Il2rgtm1Wjl/Sz), female, 6-8 weeks old. housing conditions: light/dark cycle 7-7, 21 °C and at 50% humidity

Wild animals

none

Field-collected samples

the study did not involve samples collected from the field

Ethics oversight

All animals in the experiments discussed were performed under project license (P83B37B3C), approved by the UK Home Office, and in accordance with The Francis Crick Institute animal ethics committee guidelines.

Note that full information on the approval of the study protocol must also be provided in the manuscript.
